# First evaluation of the Next-Generation Sequencing platform for the detection of HIV-1 drug resistance mutations in Belgium

**DOI:** 10.1371/journal.pone.0209561

**Published:** 2018-12-31

**Authors:** Géraldine Dessilly, Léonie Goeminne, Anne-thérèse Vandenbroucke, Francois E. Dufrasne, Anandi Martin, Benoît Kabamba-Mukabi

**Affiliations:** 1 Université catholique de Louvain (UCLouvain), Institut de Recherche Expérimentale et Clinique (IREC), Medical Microbiology Unit (MBLG), AIDS Reference Laboratory, Brussels, Belgium; 2 Université catholique de Louvain (UCLouvain), Cliniques Universitaires Saint-Luc, Clinical Laboratory Department, Brussels, Belgium; National and Kapodistrian University of Athens, GREECE

## Abstract

**Introduction:**

The WHO urges action against the threat posed by HIV drug resistance. It is well known that the sensitivity of Next-Generation Sequencing (NGS) is greater than that of Sanger Sequencing (SS). The objective of this study was to evaluate the performance of the novel NGS HIV-1 drug resistance monitoring system.

**Materials & methods:**

NGS analyses were performed on 67 plasma samples from HIV-1 infected patients using the *Sentosa* SQ HIV Genotyping Assay from Vela-Dx. This kit was used on a semi-automated Ion Torrent-based platform. Sequences were compared to those obtained by SS. Samples were analysed in the same and in separate runs. Quality controls (QC) were added to control sequencing processes of protease (PRO), reverse transcriptase (RT) and integrase (INT) regions.

**Results:**

Of the 41 analysed samples, 33 (80.5%) had identical drug resistance interpretation reports. Discrepant results were observed for eight samples. Five of them were only detected by NGS and had drug resistance mutations (DRMs) with an allelic frequency below the limit of detection of the SS method (between 6.3 to 20.5%). Two DRMs were only identified using the SS method. The sequences were similar in 98.2% of cases (counting variants as mismatches) and homologous in 99.9% if missed variants. Duplicated samples in a single run were similar in 95.7% (99.9%) of cases. Duplicated samples in two different runs were 98% (100%) homologous. QC results were manually assessed with a score of 340/340 for detection of DRMs in PRO and RT and 100% for INT sequencing.

**Conclusions:**

This is the first preliminary evaluation in Belgium employing the *Sentosa* SQ HIV Genotyping Assay. The NGS appears to be a promising tool for the detection of DRMs in HIV-1 patients and showed a higher sensitivity compared to SS. Large studies assessing the clinical relevance of low frequency DRMs are needed.

## 1. Introduction

Human immunodeficiency virus (HIV) type 1 is the agent responsible for acquired immunodeficiency syndrome (AIDS) and for the majority of HIV infections worldwide [1, 2]. Although the number of newly diagnosed cases of HIV decreases from year to year, the number of infected patients is continuing to increase with a total of 15 000 diagnosed patients in follow-up in Belgium [3] and an estimation of 36.7 millions of people living with HIV in the worldwide [4].

The current treatment for HIV consists of a combination of antiretroviral drugs (ARV), typically three drugs from two or more classes [5]. The ARV regimen for a treatment-naive patient generally consists of two nucleoside reverse transcriptase inhibitors (NRTIs) in combination with a third active ARV drug from one of three drug classes: an integrase strand transfer inhibitor (INSTI), a non-nucleoside reverse transcriptase inhibitor (NNRTI), or a protease inhibitor (PI) with a pharmacokinetic (PK) enhancer (booster) (cobicistat or ritonavir) [5].

Viral load is the most important indicator of initial and sustained response to ART and should be measured in all patients with HIV at entry into care, at initiation of therapy and on a regular basis thereafter. The level of viral load level before treatment is also an important factor in the selection of an initial ARV regimen because several currently approved ARV drugs have been associated with poor responses in patients with high baseline viral load. The goal of ARV treatment (ART) is to continually keep plasma HIV-RNA levels below the level of detection, 50 RNA viral copies/mL (c/mL), depending on the assay used [5]. Highly active antiretroviral therapy (HAART) has significantly reduced morbidity and mortality in HIV-1 infected patients [6]. However, the effectiveness of HAART can be compromised by the presence of drug resistance mutations (DRMs), resulting in virological failure. European, International AIDS society and treatment guidelines recommend ARV drug resistance testing for all HIV-1 infected patients before treatment initiation and after treatment failure. Moreover, guidelines recommend the use of genotyping in most routine clinical situations. Current genotyping can be performed below a viral load of 1000 c/mL [7, 8].

Standard genotypic drug-resistance testing in ARV-naive persons involves testing for mutations in the protease (PRO) and reverse transcriptase (RT) regions in HIV-1 polymerase (*pol*). Although reports of transmission of INSTI-resistant virus are rare, as use of INSTIs increases, the potential for transmission of INSTI-resistant virus may also increase. Therefore, when INSTI resistance is suspected, providers should supplement standard baseline genotypic resistance testing with genotypic testing for resistance to this class of drugs [5].

In clinical practice, sequencing is usually performed using Sanger Sequencing (SS) which enables the detection of drug resistance mutations (DRMs) present in at least 20–30% of the viral population to be detected [9, 10]. However, DRMs occurring less frequently, called “minority variants”, are not detected using the SS method.

Recent Ultra-Deep Sequencing (UDS) or Next-Generation Sequencing (NGS) technologies are known to have, in the majority of cases, better sensitivity and reproducibility than the SS method. NGS technologies perform millions of parallel sequencing reactions to generate many, typically short, reads per run. These technologies can detect and quantify low-frequency variants down to 5% of the total virus population [10–15].

The introduction of NGS into clinical laboratories has been slow for several reasons: (i) the impact on drug response and thus the clinical relevance of low-frequency DRMs before ART and after virological failure remains open to debate [14, 16–19], (ii) the high costs of this technology, (iii) the turnaround time and the number of samples per run.

Vandenhende M-A et al. [20] reported that low-frequency DRMs detected before ART initiation and after virological failure in patients receiving the first-line ART could increase the overall burden of resistance to PI, NRTI and NNRTI. More than two-thirds of these patients had additional low-frequency DRMs only detected by NGS. This observation is in accordance with previous studies that describe the abundance of low-frequency DRMs detected by NGS in treatment-naive patients [21] as well as in treatment-experienced patients [14, 19, 22], in some cases changing the susceptibility to the prescribed treatment.

However, a recent study by Raymond S et al., using the NGS 454 GS-FLX system, showed that drug-resistant minority variants had no impact on the virological failure of treatment-naive patients to a rilpivirine-based regimen [23].

Few studies have been conducted to compare and evaluate NGS and to determine its detection limit for HIV DRMs, to acquire expertise in performing analyses, or to collect data on which percentage of minority variants is predictive of treatment efficacy.

A few years ago, Mohamed and his team has compared HIV-1 DRMs minority variants after virological failure between SS and NGS. They demonstrated that NGS detected all mutations found by SS and identified also additional resistant variants [14].

Recently, Trabaud and his team [11] performed a prospective study in 100 clinical samples to compare SS and NGS (Roche 454 system) methods. Analyses were successful for 88% of samples regardless of HIV viral load or subtype. Setting the cut-off at 5% of minority variants for NGS seemed to be a good compromise to take into account the ability of NGS to detect low-prevalence DRMs, but without frequent reporting of mutations that are not detected by SS. With NGS, the authors identified additional PI resistance mutations. These mutations were mainly secondary mutations in treatment-naive as well as in treatment-experienced patients. Moreover, RT mutations identified only by NGS were mainly observed in treated patients and were consistent with their drug treatment history.

The *Sentosa* SQ HIV genotyping assay (Vela-Diagnostics, Germany) is a novel deep-sequencing, *in vitro* semi-automated and standardised system. The objective of this study was to evaluate this novel NGS HIV-1 drug resistance monitoring system in a population of HIV-1 infected patients representative of those followed in clinical routine. More precisely, we analysed the repeatability (intra- and inter-run), the precision and the sensitivity of this NGS workflow. We also investigated DRMs and low-prevalence mutations detected by NGS compared to the SS as the reference method with the goal to accreditate this platform in our clinical routine.

## 2. Materials and methods

### 2.1 Patients

A total of 67 plasma samples collected from 40 patients (see details in Table 1) and previously tested using the routine SS assay at the AIDS Reference Laboratory in Brussels (Université catholique de Louvain) were analysed with the Sentosa SQ HIV genotyping assay (Vela Diagnostics, Germany). Selected samples represent the HIV-1 infected patients population followed at the AIDS Reference Laboratory. This retrospective observational study does not require any agreement of an ethical committee. HIV-1 viral loads had previously been measured using the Abbott m2000 RealTime HIV assay (Abbott Diagnostics, France). The plasma specimens had been frozen and stored at -80°C until testing.

**Table 1:** Characteristics of patients population.

Characteristics	n = 40
Subtypes	
B	14
C	11
A1	4
Other	11
Viral load (c/mL)	
Average	72442
Median	699
Range	300−10^6^
ART treatment	
None	19
Interruption	4
NRTI/PI	6
NRTI-NNRTI/INI	8
NRTI/PI/INI	2

ART: antiretroviral treatment, NRTI: nucleoside reverse transcriptase inhibitor, PI: protease inhibitor, NNRTI: non-nucleoside reverse transcriptase inhibitor, INI: integrase inhibitor

### 2.2 Sanger sequencing

Viral RNA was extracted from 1 mL of plasma sample at baseline and at virological failure using MagNA Pure Compact Nucleic Acid Isolation Kit I Large volume on MagnaPure compact automate (Roche Diagnostics, Belgium). A nested PCR was performed. The sequences of the amplification primers (internal PCR) were represented in the Table 2.

**Table 2:** Sequences of the amplification primers.

Fwd-PRORT-I1	5’-CCAGARCAGRCCAGAGCCAACAGCCCCA-3’
Fwd-PRORT-I2	5’-AGAGCCAACRGCCCCACC-3’
Rev-PRORT-I1	5’-TTCTGCTATTAATTCYTTTGCTGG-3’
Rev-PRORT-I2	5’-TTCTGCTAYTAAGTCTTTTGATGGRT-3’
Rev-PRORT-I3	5’-TTCAGCTATYAAGTCTTTTGATGG-3’
Fwd-INT-I1	5’TTCRGGATYAGAAGTAAAYATAGTAACAG-3’
Rev-INT-I1	5’TCCTGTATGCARACCCCAATATG-3’

FWD: forward primer; REV: reverse primer; PRORT: protease and reverse transcriptase; INT: integrase; I: internal PCR

The sequencing was performed using the big dye terminator v3.1 ready reaction mix on ABI3500 automated sequencer (Life technologies, Belgium). The PRO, RT and INT sequences were determined using SS according to the Stanford consensus method. Sequences were proofread using the SmartGene HIV module (Lausanne, Switzerland) and the sequence editing was confirmed manually. Mutations compared to HIV-1 Consensus B reference were reported with this module as well as the drug resistance interpretation.

### 2.3 NGS using the Sentosa SQ HIV genotyping assay

The assay was performed according to the manufacturer’s instructions (Vela-Dx, Germany). The Sentosa SQ HIV genotyping assay is reserved for the detection of HIV DRMs in PRO, RT and INT regions from plasma samples. The NGS workflow automates nucleic acid extraction from 730uL plasma samples and PCR set-up (Sentosa SX101), followed by an off-board PCR amplification (Veriti 96-well thermal cycler, Applied Biosystems). The workflow continues with normalisation, enzymatic shearing, purification and adapter ligation (Sentosa SX101). Finally, the two last steps consist of template preparation and sequencing, based on ion torrent technology (Sentosa ST101 and SQ301).

The assay contains several controls: a system control (SC), a positive and a negative control. The SC is added as a sample to control the entire workflow from RNA extraction to sequencing data analysis (functions as a positive control), as well as ensuring the absence of contamination in the workflow (functions as a negative control). An internal control (IC) is also added to each sample at the beginning of the extraction. The IC is a positive control for both nucleic acid extraction and library preparation steps. The third control is the Ion Dx CF-1 control, added during the emulsion. This control checks the steps of template preparation and sequencing.

### 2.4 Reference samples

A standard panel, HIV-1 drug resistance EQA (QCMD-2016) (n = 5), was purchased form Quality Control for Molecular Diagnostics (Glasgow, Scotland, UK). These external quality control samples were tested to assess HIV-1 protease and reverse transcriptase sequencing performances. Consensus sequences provided by QCMD were obtained by aligning the sequences of all participating laboratories, mainly European laboratories in the HIV field. A sample from INSTAND-2015 (n = 1) (Instand, Düsseldorf, Germany) external quality control was tested to control the integrase region. We also tested an 8E5 HIV positive cell line, derived from LAV-infected cells, a CD4^+^ CEM-derived human T-cell line (obtained from the NIH AIDS Reagent Program, Division of AIDS, NIAID, NIH) as an internal quality control (IQC) (diluted in HIV-negative plasma).

### 2.5 Study design

The HIV-1 subtype and drug resistance analysis were determined in 67 clinical specimens and standard panel members by in house SS assay. These results were compared with those generated by Sentosa SQ HIV genotyping assay. Samples were previously genotyped on ABI3500 automated sequencer (Life Technologies, Belgium) and sequences were analysed with IDNS-HIV1 SmartGene module (Lausanne, Switzerland).

### 2.6 Data interpretation

FASTA sequences were compared using raw data from Sentosa-Vela.

DRMs (as well as reads, coverage) and drug resistance reports were compared by exporting raw FASTQ sequences from Vela and analysing them with the specific UDS-HIV SmartGene module, IDNS-ASP (Lausanne, Switzerland). Reads were filtered to eliminate low quality reads (elimination of short sequences, excision of primers sequences alignments). An ambiguity filter was used in order to remove uncertain nucleotides. This process is automated and the final alignment can be finally visualised.

An interpretation of the genotypic drug resistance report, by using the Stanford 8.4.0 algorithm, was generated with detected mutations and their percentages. The nucleotide ambiguity filter was set at 0.5% and the threshold for interpretation of resistance at 5% when the position was covered by at least 50 reads.

### 2.7 Statistical analysis

The proportions of mutations detected by NGS and SS, categorised according to the percentage of minority variants population, were compared using the ANOVA-test (p<0.05).

## 3 Results

### 3.1 Repeatability

Intra-assay repeatability was evaluated by analysing patient samples twice in a single run (n = 3) (Table 3). Inter-assay repeatability was evaluated by analyzing patient samples twice in two different runs (n = 6) (Table 4).

**Table 3:** Intra-assay repeatability.

Sample * (vL c/mL)	All mutations identified with SS and NGS	DRMs identified with SS and NGS	DRMs identified with NGS (%) in one duplicate	Drug resistance reports with NGS in one duplicate
V15-1676 (2229)	PRO: 11I, 13V, 16E, 17E, 20R, 35D, 36I, 37D, 60E, 62V, 63T, 69K, 89I, 93L RT: 35T, 60I, 107S, 122E 123G, 142V, 177E, 178L, 200A, 202V, 207A, 211K 214L, 244V, 245Q, 272P, 277R, 282I, 286A, 291D, 292I, 293V, 310I, 312G, 317A, 335D, 356K, 359S, 379C, 379G, INT: 17N, 20K, 24N, 31I, 68V, 72V, 74I, 112V, 124A, 125A, 127R, 134D, 136Q, 136R, 138D, 188R, 201I, 218L, 221H, 222K, 234I, 256E, 265V, 268L	/	RT: M41L (31.9%)	Low-level resistance to AZT, D4T Potential low-level resistance to DDI
V16-2178 (1996)	PRO: 10V, 15V, 20R, 35D, 36I, 41K, 57K, 63T, 65D, 77I, 89M RT: 14S, 35T, 39A, 40D, 60I, 67N, 69D, 70R, 98G, 106G, 123E, 135L, 162H, 173T, 174K, 195L, 200A, 203D, 207A, 211K, 219Q, 228H, 245Q, 248D, 272P, 272S, 291D, 292I, 293V, 294T, 297T, 326V, 334E, 345I, 356K, 359S, 366R, 377M, 379C, 379G INT: 11D, 13D, 14R, 20K, 101I, 112R, 124S, 125A, 136Q, 146H, 158F, 193E, 201I, 205S,218I, 234V, 256E, 279G, 283G	RT: 67N, 69D, 70R, 98G, 219Q	RT: L100V (6.3%)	Different score of resistance to all NNRTI, Low-level resistance to ETR
V17-1123 (9480)	PRO: 19I, 41K, 45R, 46I, 53L, 60E, 62V, 63P, 77I, 85V, 92E, 92K RT: 49R, 50V, 60I, 83K, 162H, 162Y, 177N, 207K, 211T, 272P, 275R, 278N, 281R, 286A, 297K, 333E, 357V, 379C, 379G INT: 124A, 124N, 154I, 182V, 201I, 256E	PRO: 46I, 53L	/	/

VL: viral load, NGS: Next-Generation Sequencing, DRMs: Drug Resistance Mutations, PRO: protease, RT: reverse transcriptase, INT: integrase, AZT: Zidovudine, D4T: stavudine, DDI: didanosine, NNRTI: non-nucleoside reverse transcriptase inhibitor, ETR: etravirine

* patient sample was evaluated twice

**Table 4:** Inter-assay repeatability.

Sample * (vL c/mL)	All mutations identified with SS and NGS	DRMs identified SS and NGS	DRMs identified with NGS (%) in one duplicate	Drug resistance reports with NGS in one duplicate
V16-7289 (1550)	PRO: 13V, 14R, 15V, 20I, 36I, 39Q, 63T, 69K, 82I, 89M RT: 21I, 35T, 50V, 60I, 102Q, 122E, 123N, 142V, 162N, 173T, 174K, 177E, 178L, 200A, 207D, 211K, 245Q, 250E, 272P, 283I, 292I, 293V, 297A, 326V, 329V, 334L, 335D, 346Y, 356K, 357R, 358G, 359A, 359S, 371V, 379C, 379G INT: 14R, 31I, 50I, 63I, 72V, 74I, 101I, 112V, 124A, 125A, 134D, 135V, 136T, 181L, 201I, 206S, 218S, 234I, 254T, 254insert, 255insert, 255K, 255N, 256E, 283G	/	RT: D67N (6.9)	Low-level resistance to AZT, D4T
V16-7338 (91971)	PRO: 12S, 14R, 15V, 19I, 37S, 41K, 63M, 69K,77I, 82I, 89M, 93L RT: 35T, 36A, 39E, 48T, 69A, 104R, 162C, 173A, 174K, 177E, 178M, 200A, 203D, 207D, 207N, 210S, 211K, 245Q, 248D, 248N, 250E, 251N, 272P, 275Q, 291D, 292I, 293V, 294T, 329L, 334D, 335D, 356K, 359N, 376S, 377V, 379C, 379G INT: 11D, 24N, 25E, 31I, 100Y, 101I, 112V, 112I, 124A, 125A, 136Q, 162V, 163A, 163Q, 201I, 234I, 278A, 283G, 289Q	/	/	/
V17-0688 (3685)	PRO: 12A, 14R, 15V, 16A, 16E, 19I, 35D, 36I, 37D, 51E, 57K, 60E, 61D, 61E 63T, 65D, 67E, 69K, 72T, 77I, 89M RT: 4T, 6D, 8I, 35T, 35E, 35K, 39M, 40D, 49R, 83K, 99, 103N, 122E, 123N, 137S, 138A, 142T, 159V, 162H, 173T, 177E, 178L, 179I, 184V, 196E, 200E, 202V, 207E, 207N, 211K, 225H, 237E, 238T, 245Q, 248D, 250E, 272P, 277R, 286A, 291D, 292I, 293V, 294S, 297A, 297K, 301F, 311R, 314M, 324E, 329V, 334E, 335S, 335D, 356K, 357R, 357T, 359T, 366R, 371V, 377V, 379C, 379G INT: 11D, 14R, 17N, 21T, 31I, 45V, 72V, 91T, 101I, 111R, 112V, 119P, 122I, 124A, 125A, 134D, 136Q, 157Q, 160T, 160N, 167E, 173R), 201I, 203M, 208L, 212L, 234V, 256E, 260I, 283G	RT: 103N, 138A, 184V, 225H, 238T INT: 157Q	/	/
V17-1006 (31090)	PRO: 13V, 14R, 37S, 63P, 72V, 77I RT: 36D, 118I, 123E, 135R, 142V, 162C, 179I, 196E, 211G, 215E, 245K, 292I, 293V, 356K, 357R, 357I, 360T, 366R, 370D, 377I, 377L, 379C, 379G INT 17N, 45V, 45I, 100Y, 101I, 111R, 112I, 119P, 163A, 201I, 206S, 211R, 219N, 253E, 255G, 256E, 279G, 288G	215E	/	/
V17-1092 (975)	PRO: 13V, 14R, 20I, 36I, 37D, 38I, 41K, 63P, 64V, 67Y, 69K, 89I RT: 27S, 35T, 36D, 60I, 68N, 135V, 162A, 173T, 174E, 177E, 178V, 200A, 207E, 245Q, 272S, 274V, 277R, 286A, 291D, 293V, 294T, 322T, 326R, 328D, 335D, 356K, 357K, 359S, 366R, 369A, 370K, 371V, 375V, 379C, 379G INT: 3N, 14R, 21T, 39N, 65R, 68V, 84M, 101I, 112V, 124A, 125A, 134N, 135V, 136T, 201I, 206S, 208M, 265V, 283G	/	/	/
V17-1123 (9480)	PRO: 19I, 41K, 45R, 46I, 53L, 60E, 62V, 63P, 77I, 85V, 92E, 92K RT: 49R, 50V, 60I, 83K, 162H, 162Y, 177N, 207K, 211T, 272P, 275R, 278N, 281R, 286A, 297K, 333E, 357V, 379C, 379G INT: 124A, 124N, 154I, 182V, 201I, 256E	46I, 53L	/	/

VL: viral load, NGS: Next-Generation Sequencing, DRMs: Drug Resistance Mutations, PRO: protease, RT: reverse transcriptase, INT: integrase, AZT: Zidovudine, D4T: stavudine

* patient sample was evaluated twice

All real mismatches were at the end of the sequences and as a consequence did not affect the drug resistance reports.

All mutations (non-DRMs and DRMs) were correctly identified with SS and NGS methods. The NGS has identified some DRMs, who were not identified by SS method, in only one of the duplicates (see Tables 3 and 4). The recommended criteria of Vela-Dx for the accurate detection of targeted mutation are a variant frequency of 20% at 1000 c/mL or of 5% at 4000 c/mL and were fulfilled for two of three samples.

The criteria of Vela-Dx were not fulfilled for only one sample. Indeed, despite a viral load >1000 c/mL and correct coverage at this position, a mutation presents at 32% (>20%) was not detected in one of the duplicates. This discordant result (for DRMs and drug resistance reports) is reported in the section 3.6 and in the discussion section.

8E5 IQC was used to evaluate the reproducibility. Indeed, one mutation was followed in each region; PRO, RT and INT regions (Table 5). More precisely, the mutations (non-DRMs) PRO18E, RT379C and INT265V were followed. For each mutation, a variation of +/- 5% around the average was accepted. The three mutations were identified within acceptability limit.

**Table 5:** 8E5 internal quality control reproducibility.

Sample	Reads	% PRO 18E	% CL PRO 18E	% LCL PRO 18E	% UCL PRO 18E	% RT 379C	% CL RT 379C	% LCL RT 379C	% UCL RT 379C	% INT 265V	% CL INT 265V	% LCL INT 265V	% UCL INT 265V
**IQC-1**	47034	5,5				14,0				93,0			
**IQC-2**	86736	3,0				15,4				94,9			
**IQC-3**	211696	1,9				15,2				92,8			
**IQC-4**	112359	2,9				16,4				95,2			
**IQC-5**	76572	2,8				17,3				94,4			
**IQC-6**	165896	1,9				16,1				96,5			
**IQC-7**	247282	2,9				15,2				97,1			
**IQC-8**	80727	2,1				16,2				94,2			
**IQC-9**	102860	1,8				19,0				95,6			
**IQC-10**	144248	1,9	2,7	-2,3	7,7	16,1	16,1	11,1	21,1	94,7	94,8	89,8	99,8

IQC: internal quality control, PRO: protease, RT: reverse transcriptase, INT: integrase, CL: average percentage of the mutation: LCL: lowest average percentage of the mutation (-5%), UCL: uppest average percentage of the mutation (+5%)

### 3.2 Precision

A total score of 340/340 was obtained for the QCMD-2016 (01–05) external quality controls for HIV-1 PRO and RT regions (Table 6). The QCMD proficiency program assigns one point for an identical codon to the consensus sequence and one point if at least one amino acid is identical to the consensus sequence. For the INSTAND-2015 external quality control, the INT sequence obtained using NGS was 100% identical to that obtained using the SS method (Table 7).

**Table 6:** QCMD precision.

Sample	SS Clade	NGS Clade	Score	Difference: SS nt(aa)—NGS nt(aa)
ENVA16-01	C	C	68/68	RT103: AAA (K)—RAA (K/E) RT190: GGA (G)—GRA (G/E)
ENVA16-02	C	C	68/68	None
ENVA16-03	B	B	68/68	RT179: GTT (V)—GYT (V/A)
ENVA16-04	C	C	68/68	PRO71: GCT (A)—RCT (A/T)
ENVA16-05	D	D	68/68	PRO71: GCT (A)—RCT (A/T) RT103: AAA (K)—RAA (K/E)

SS: sanger sequencing, NGS: next-generation sequencing, nt: nucleotide, aa: amino-acid, PRO: protease, RT: reverse transcriptase

**Table 7:** INSTAND precision.

Sample	SS clade	NGS clade	Criteria	SS Result	Success rate (number of participating labs)	NGS result
384–003 (INSTAND)	B	B	Number of different nt by 100 nt of the INSTAND consensus sequence	<6,5	100% (29/29)	0
			Determination of DRMs	N155H G163R S230N	100% (30/30)	N155H G163R S230N
			Resistance interpretation	DTG: S/I EVG: R RAL: R	100% (31/31)	DTG: S/I EVG: R RAL: R

SS: sanger sequencing, NGS: next-generation sequencing, nt: nucleotide, DRMs: Drug Resistance Mutations, DTG: dolutegravir, EVG: elvitegravir, RAL: raltegravir

Three of five QCMD-2016 samples presented resistance mutations (Table 8). All DRMs were correctly identified with SS and NGS methods.

**Table 8:** Interpretation report of QCMD resistant samples.

Sample	Clade	DRMS identified with SS and NGS (%)	Drug resistance report
ENVA16-01	C	PRO: 46I (99.5), 54V (98.6), 82A (91.8) RT: 184V (100), 190E (7.7)	High level: 3TC, FTC, EFV, NVP, RPV, ATV, FPV, IDV, LPV, NFV, SQV Intermediate: ETR, FPV Low level: ABC, TPV Potentiall low-level: ddI
ENVA16-02	C	RT: 41L (99.4), 44D (39), 67N (99.8), 69D (100), 98G (100), 184I (100), 188L (100), 190A (100), 210W (100), 215Y (94.3),	High level: 3TC, ABC, AZT, D4T, ddI, FTC, TDF, EFV, NVP, RPV Intermediate: ETR
ENVA16-04	C	RT: 67N (97.3), 70R (99.1), 184V (98.4), 219Q (100)	High level: 3TC, ABC, FTC Intermediate: AZT, D4T, ddI Low level: TDF

PRO: protease, RT: reverse transcriptase, 3TC: lamivudine, FTC: emtricitabine, EFV: efavirenz, NVP: nevirapine, RPV: rilpivirine, ATV: atazanavir, FPV: fosamprenavir, IDV: indinavir, LPV: lopinavir, NFV: nevirapine, SQV: saquinavir, ETR: etravirine, ABC: abacavir, TPV, tipranavir DDI: didanosine, AZT: zidovudine, D4T, stavudine, TDF: tenofovir

Moreover, 8E5 IQC was diluted successively 1/2, 1/5 and 1/10 (serial dilutions) and compared to SS results. All mutations found in SS were correctly identified with NGS. We obtained 100% of sequences homology, with no mismatch. Moreover, there was no difference in resistance reports between SS and NGS methods (date not shown).

### 3.3 Analytical sensitivity

The limit of detection (LOD) was estimated with the 8E5 IQC. IQC was diluted to obtain a viral load of 200 and 100 c/mL (Table 9), with as the acceptance criteria of at least 95% of success rate. No acceptable result was obtained for a viral load of 100 c/mL (n = 2). The sequencing of INT region was failed. The coverage for PRO and RT regions were in warning status with very low values (42–539). 50% of success rate for a viral load of 200c/mL was obtained (n = 2). INT region was successfully sequence but the coverage for PRO and RT regions appeared in warning status (94–593).

**Table 9:** Analytical sensitivity of internal quality control and samples.

Sample	Viral load (c/mL)	Subtypes	Number of reads PR/RT/INT	Coverage status (value)
IQC-200_1	200	B	40865	-Passed INT -Warning PRO & RT (94–470)
IQC-200_2	200	B	56708	-Passed INT -Warning PRO & RT (133–593)
IQC-100_1	100	B	71340	-Warning PRO & RT (129–539) -Failed INT
IQC-100_2	100	B	7513	-Warning PRO & RT (42–378) -Failed INT
V16-1757 Dilution 1/10	285	G	161246	-Passed
V17-2597	300	C	25745	-Warning PRO (510 to 996) -Warning RT (421 to 663) -Warning INT (730 to 992)
V16-2001	494	A1	61429	-Warning PRO (547–878)
V16-1638	620	A1	96226	-Passed
V16-0052	669	B	56186	-Warning all INT (301–742)
V16-4015	724	CRF02AG	31661	-Warning PRO (406–990) -Warning RT (513–982) -Warning INT (566–972)
V17-0521	778	A1U	46352	-Warning PRO (455–915) -Warning INT (576–929)
V11-6747 Dilution 1/2	808	C	111983	-Warning INT (683–960)
V16-0300	861	D	74007	-Passed
V17-1092	975	CRF02AG	73961	-Warning PRO (852–983)

IQC: internal quality control, PRO: protease, RT: reverse transcriptase, INT: integrase, Passed: coverage>1000, Warning: coverage between 50 and 1000, Failed: coverage <50

Based on this IQC analysis, patient samples (n = 10) with a viral load between 285 to 975 c/mL (different subtypes, ARV therapy naive or experienced) were selected to establish a LOD. The viral loads 3-fold lower than analytical sensitivity established by Vela Dx (1000 c/mL) were evaluated. All samples were appropriately amplified and sequenced (in term of number of reads and coverage) and the LOD was set at 300 c/mL (Table 9). The warning status appeared for some samples for which, a specific attention has been made in checking at each position.

### 3.4 Comparison between SS and NGS methods

**Fig 1:**
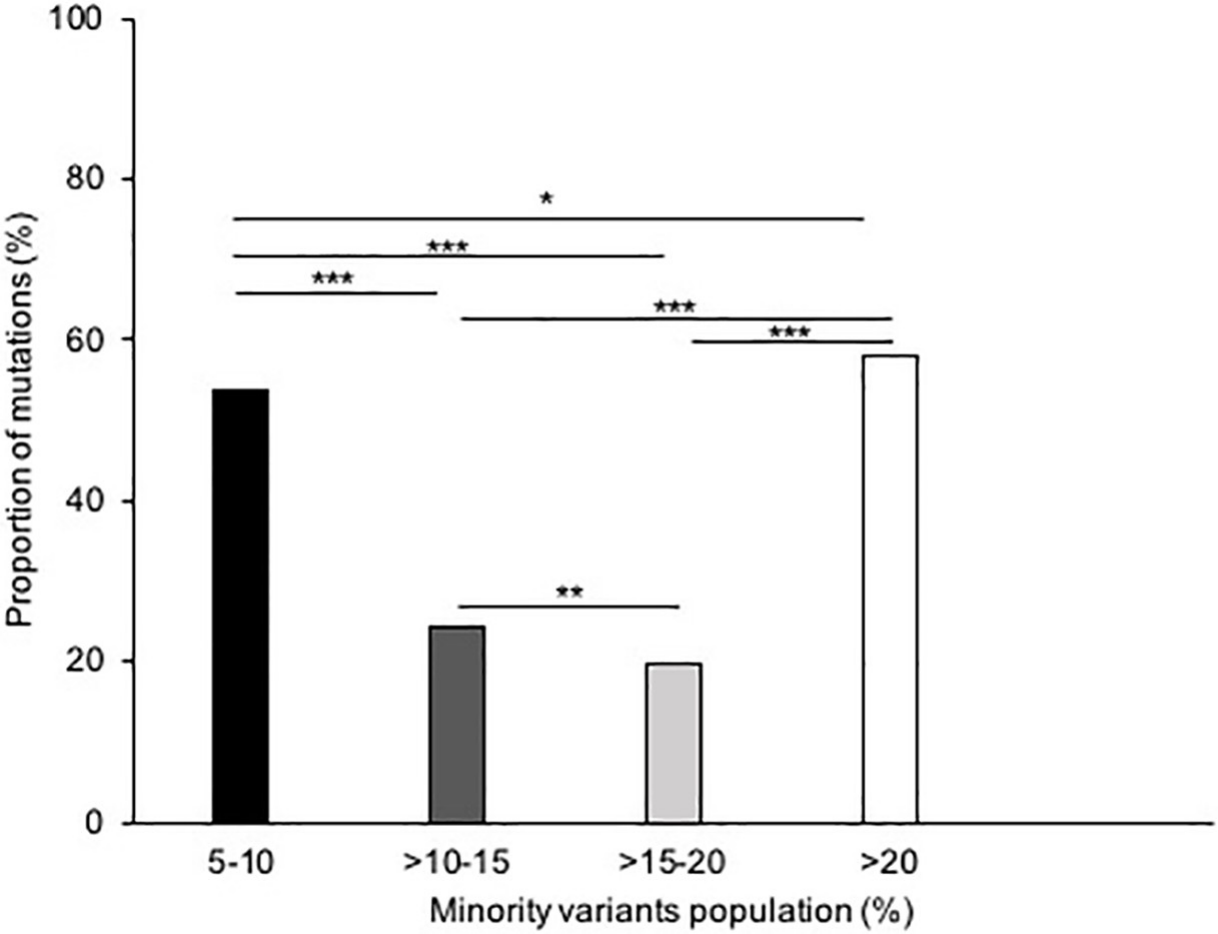
Proportion of minority variants detected by Next-Generation Sequencing. Proportion of minority variants detected by Next-Generation Sequencing. The proportion of mutations (%) is reported by minority variants population detected (%). In black: between 5 and 10%, in dark grey: between 10 and 15%, in light grey: between 15 and 20% and in white: >20%. *p<0.05, **p<0.01, ***p<0.001.

This comparison allows the evaluation of the ability of detecting a mutation in one assay compared the other. A total of 614 differences were identified in the 41 samples (40 HIV-1 infected patients and one IQC) (Table 10), using the NGS method compared to the SS method, among which 257 (41.86%) were minority variants (between 5 to 20%). More precisely (Fig 1), 138 differences (53.7%) were present with a frequency between 5 and 10%, 68 (26.46%) at >10 to 15%, 51 (19.84%) at >15 to 20% and 357 (58.14%) samples had mutations with a frequency >20%. Moreover, 530/614 (86.3%) of these differences were present in the RT region, 46/614 (7.5%) in the PR region and 38/614 (6.2%) in the INT region.

**Table 10:** Comparison of mutations between Sanger Sequencing and Next-Generation Sequencing.

	NGS	
**SS**	0	1
0	/	614
1	60	1516

SS: sanger sequencing, NGS: next-generation sequencing, 0: absence of mutation 1: Presence of mutations

There were 60 differences identified using the SS method compared to the NGS method (Table 10). These differences were classified into three categories. In the first category, 46/60 (76.87%), we included sequences with a high background interference at the baseline (background sequencing signal), manual correction mistakes, only one strand of reads (forward or reverse) or when the mutation was weakly present. In the second category, 9/60 (18.33%), the observed differences reflected that NGS distinguished cases when combinations of a codon were really present compared to SS. NGS could discern exact mixtures of more than two amino acids, whereas SS could not. Finally, the last category, 5/60 (15%), represented the situation where the mutation was indeed present.

We detected 1516 mutations that were identified with both methods (SS and NGS), 357 of which (23.55%) were minority variants (frequency between 5 and 20%).

**Fig 2:**
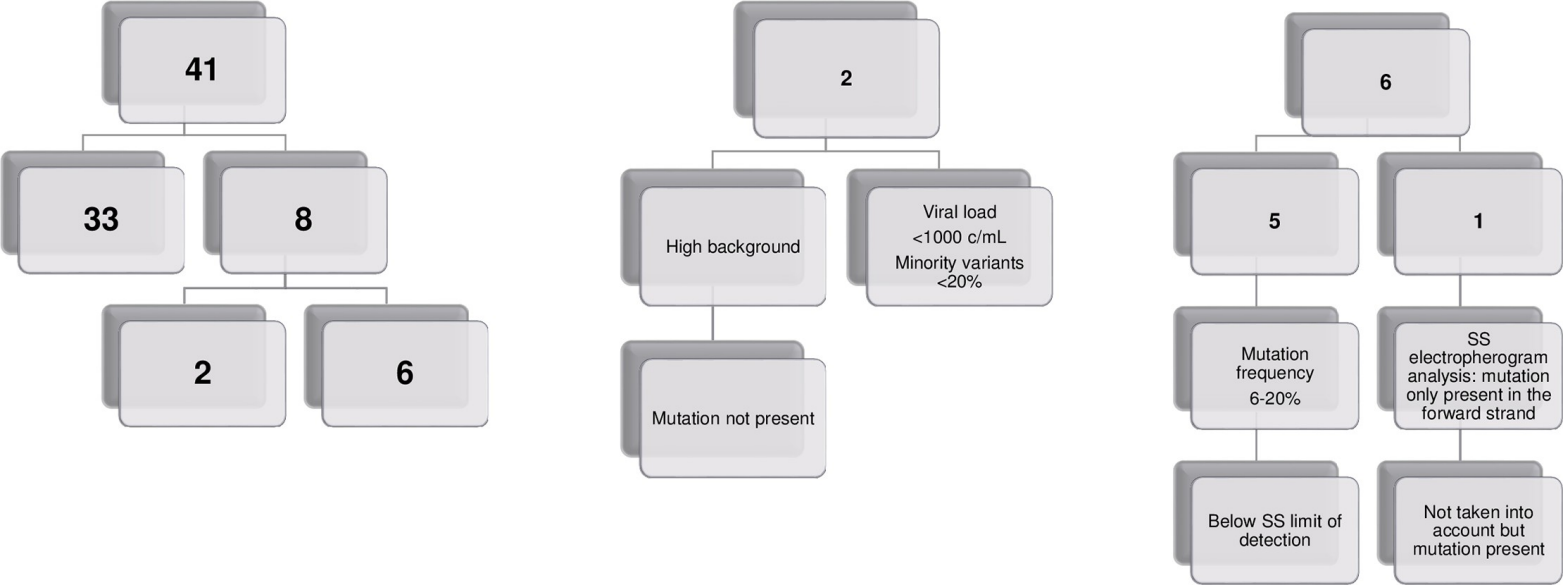
a-b & -c: Drug resistance interpretation reports with the resistance of drug mutations between Sanger Sequencing and Next-Generation Sequencing. Drug resistance interpretation reports with the resistance of drug mutations between Sanger Sequencing and Next-Generation Sequencing. Comparison of drug resistance interpretation reports (in purple) and Drug Resistance Mutations (in red) between Sanger Sequencing and Next-Generation Sequencing. SS: Sanger Sequencing.

Drug resistance interpretation from the SS and NGS data was compared using the Stanford 8.4.0 algorithm (Fig 2A, 2B & 2C). Among the 41 samples, 33 (80.5%) had identical drug resistance interpretation reports. Discrepant results were obtained for 8 samples. Five of these samples had DRMs (minority variants) with an allelic frequency below the LOD of the SS method (6.3 to 20.5%) and are represented in the Table 11.

**Table 11:** Drug resistance mutations and reports obtained by Next-Generation Sequencing.

Sample	DRMs (region) (%)	Difference in resistance reports
V11-6747	46I (PRO) (6.8)	-Potential low-level: ATV, FPV, IDV, LPV, SQV -Intermediate: NFV
V15-6348	20T (PRO) (20.5)	-Low-level: NFV
V16-2178	100V (RT) (6.3) (associated to 98G)	-Low-level: ETR -Intermediate: RPV -Low-level: ETR -High level: NVP
V16-6496	69D (RT) (7.1) 70E (RT) (10)	-Potential low-level resistance: 3TC, FTC -Low-level resistance: ABC, D4T, TDF -Intermediate resistance: DDI
V17-0297	179D (RT) (6.1) (associated to 98G)	-Low-level resistance: EFV, ETR, RPV

DRMs: drug resistance mutations, PRO: protease, RT: reverse transcriptase, ATV: atazanavir, FPV: fosamprenavir, IDV: indinavir, LPV: lopinavir, SQV: saquinavir, NFV: nevirapine, ETR: etravirine, RPV: rilpivirine, NVP: nevirapine, 3TC: lamivudine, FTC: emtricitabine, ABC: abacavir, D4T: stavudine, TDF: tenofovir, DDI: didanosine, EFV: efavirenz

One mutation was only identified by NGS at 66.2%, the 138A, conferring resistance to Raltegravir (RAL), Elvitegravir (EVG) and Dolutegravir (DTG). The analysis of electropherograms revealed that this mutation was only present in the forward strand and therefore was not taken into account in the interpretation. The analysis was repeated a second time (undiluted) and the mutation was confirmed.

Two mutations were only identified by the SS method. The first DRM was the 263K, conferring resistance to Raltegravir (RAL), Elvitegravir (EVG) and Dolutegravir (DTG). The analysis of SS electropherograms revealed that the background was high. The analysis was repeated (undiluted and diluted 1/10) and in both cases this mutation was not really present. Moreover, the importance of this DRM remains unclear according to the interpretation algorithms. The second DRM was the 103N (conferring resistance to Efavirenz and Nevirapine), the SS analysis showed that this mutation was indeed present. At this time, the possible explanation was the non-detection of a minority variant <20% for a viral load <1000 c/mL.

In the 8 discrepant results, 6/8 (75%) were RT mutations, with a 50:50 proportion of NRTI/NNRTI.

### 3.5 FASTA sequence comparison between SS and NGS

FASTA sequences using SS and NGS were compared for 40 samples and 6 external quality controls (see details in Table 12).

**Table 12:** Comparison of FASTA sequences between Sanger Sequencing and Next-Generation Sequencing.

Gene	PRO-RT & INT (n = 57)		
Statistical parameter evaluated	min	X	max
Length of sequence (pb)	1088	1385	1596
Gaps	174	240	678
Total mismatches *	0	21	69
Real mismatch	0	1	4
% of homology *	92.0	98.2	100
% of real homology	99.7	99.9	100

PRO: protease, RT: reverse transcriptase, INT: integrase, min: minimum of values, X: average, max: maximum of values

* counting minority variants not found as mismatching

We obtained some gaps, corresponding to the difference in length of sequences, with the average of 240 pb. Total mismatches, counting minority variants not found with SS and NGS, have an average of 21. Moreover, we obtained 99.9% of real sequence homology and one real mismatch.

### 3.6 Minority variants

The first parameter evaluated was fidelity and we analysed low-prevalence DRMs. The second parameter investigated was the presence of lower concentration DRMs (IQC and samples diluted). In both cases, we analysed low-prevalence DRMs that were undetected in one of the duplicates (Table 13).

**Table 13:** Evaluation of low-prevalence Drug-Resistance Mutations by Next-Generation Sequencing.

Sample	Viral load (c/mL)	Discrepant DRM	% 1	Coverage 1	% 2	Coverage 2
V15-1676	2229	RT: 41L	31.9	3060	0	1759
V16-2178	1996	RT: 100V	6.3	9626	0	3863
V16-7289	1550	RT: 67N	0	478	6.9	5922
V17-0688	3685	RT: 238T	8.8	4743	0	7690
V11-6747	1616	RT: 184I	28.7	2536	0	1034
V11-6747 Dilution 1/2	808	PRO: 46I	0	3649	6.8	561

DRM: Drug Resistance Mutation, PRO: protease, RT: reverse transcriptase, %1: percentage in the first duplicate, %2: percentage in the second duplicate

The criteria of Vela-Dx were not fulfilled for only one sample. Indeed, despite a viral load >1000 c/mL and correct coverage at this position, a mutation presents at 32% (>20%) was not detected in one of the duplicates. The analysis of the Bam files (raw data from the sequencer) by Vela-Dx revealed that this sample had at least three virus subpopulations. In the duplicate in which the mutation could not be detected, only two subpopulations could be amplified. In addition, this specific mutation was not identified with SS method.

Vela Dx criteria were fulfilled for other samples evaluated. Indeed, DRMs present at > 20% were detected for viral loads of 1000 c/mL, those >5% and <20% were detected randomly for viral loads below < 4000 c/mL.

## 4. Discussion

ARV drug resistance testing is recommended for all HIV-1 infected patients before treatment initiation and after treatment failure [7, 8]. In clinical practice, ARV drug resistance is assessed using the SS reference method, which can detect DRMs that are present in at least 20–30% of the viral population [9]. However, DRMs that are present below these values and called “minority variants”, are not detected using the SS method. NGS technologies allows the detection of low frequency HIV-1 DRMs and have potential advantages in improving patient follow-up.

The *Sentosa* SQ HIV genotyping assay by Vela-Dx is a new, automated NGS-based assay that is user friendly without specialised skills and is standardised. The present study provided an evaluation of this novel NGS HIV-1 drug resistance monitoring system in HIV-1 infected patients and compared it with the SS reference method. This evaluation provided interpretable data for samples regardless of viral loads, virus subtypes or patient history.

The LOD of the NGS sentosa assay was established at 300 c/mL. This limit was three-fold lower compared to the analytical sensitivity established by Vela-Dx (1000 c/mL).

Moreover, and in accordance to several publications [9, 10], we can confirm that the sensitivity of the NGS assay is higher than that of the SS method.

The criteria of Vela-Dx were not fulfilled for one discordant result concerning the intra-run repeatability. Indeed, despite a viral load >1000 c/mL and correct coverage at this position, a mutation presents at 32% (>20%) was not detected in one of the duplicates.

The analysis of the raw data from the sequencer revealed that this sample had at least three virus subpopulations. In the duplicate in which the mutation could not be detected, only two subpopulations could be amplified. In addition, this specific mutation was not identified with SS method.

The comparison between the two methods (SS and NGS) demonstrated that nearly half (41.86%) of the differences in the mutations identified by NGS were minority variants (occurring at frequencies of 5 to 20%). Only 23.55% of mutations were minority variants that were detected by both methods, highlighting that NGS detects twice as many minority variants, as does SS. Moreover, the majority of these mutations (86.3%) were located in the RT region.

A total of 60 mutations were only found with the SS reference method. The detection of most of these (76.87%) can be explained by the fact that the SS method remains a subjective expertise test, especially when the quality of sequences is poor, which may lead to divergent results. A small proportion of these differences (15%) may be explained by the co-existence of variants, generating SS electropherograms that can often be confusing. NGS can distinguish combinations where a codon is really encoded and can discern exact mixtures of more than two amino acids, which is not possible with SS.

The comparison of DRMs, by interpretation of drug resistance reports, confirmed that NGS more frequently detected minority variants, 75% of which were present in the RT region. This observation is in accordance with several studies that describe the abundance of low-frequency DRMs detected by NGS in treatment naive patients [21] as well as in treatment-experienced patients [14, 19, 22]. Furthermore, RT DRMs identified only by NGS were mainly identified in treated patients and were consistent with their drug treatment history.

Among the analysed samples, 80.5% had identical drug resistance reports by NGS and SS methods. Discrepant results were obtained for 8 samples. Five of these samples presented minority variants with an allelic frequency below the limit of detection of the SS method. One mutation was only identified by NGS with an allelic frequency of 66.2%, the RT 138A. The analysis of electropherograms revealed that this mutation was only present in the forward strand and therefore was not taken into account in the interpretation. Two mutations were only identified by the SS. The first DRM was the INT 263K. The analysis of electropherograms revealed that the background was high (background sequencing signal) and this mutation was not really present. The second DRM was the RT 103N, the SS analysis showed that this mutation was indeed present. At this time, the most probable explanation was a problem linked to this specific sample.

At the 5% threshold for reporting minority variants, NGS appeared to attain an increased sensitivity for detecting low-frequency DRMs without compromising sequence accuracy for viral load >1000 c/mL.

Moreover, using this NGS assay, we identified novel DRMs that affected the HIV-1 drug resistance interpretation report and may have a clinical impact.

This study has some limitations including that the platform was evaluated using a limited number of retrospective HIV-1 patient samples and discordant results were observed for minority variant DRMs and with low viral load.

In conclusion, this is the first clinical evaluation of the *Sentosa* SQ HIV Genotyping Assay in a clinical laboratory in Belgium. The NGS appears to be a promising tool for the detection and quantification of DRMs in HIV-1 infected patients. Use of NGS for resistance genotyping can provide useful information in routine clinical practice. Further studies assessing the clinical relevance of low-prevalence DRMs are needed.
