# Correction: First evaluation of the Next-Generation Sequencing platform for the detection of HIV-1 drug resistance mutations in Belgium

**DOI:** 10.1371/journal.pone.0211587

**Published:** 2019-01-25

**Authors:** Géraldine Dessilly, Léonie Goeminne, Anne-thérèse Vandenbroucke, François E. Dufrasne, Anandi Martin, Benoît Kabamba-Mukadi

The fourth author's name is spelled incorrectly. The correct name is: François E. Dufrasne. The sixth author’s name is also spelled incorrectly. The correct name is: Benoît Kabamba-Mukadi. The correct citation is: Dessilly G, Goeminne L, Vandenbroucke A-t, Dufrasne FE, Martin A, Kabamba-Mukadi B (2018) First evaluation of the Next-Generation Sequencing platform for the detection of HIV-1 drug resistance mutations in Belgium. PLoS ONE 13(12): e0209561. https://doi.org/10.1371/journal.pone.0209561

## References

[1] Dessilly G, Goeminne L, Vandenbroucke A-t, Dufrasne FE, Martin A, Kabamba-Mukabi B (2018) First evaluation of the Next-Generation Sequencing platform for the detection of HIV-1 drug resistance mutations in Belgium. PLoS ONE 13(12): e0209561 10.1371/journal.pone.0209561 30596682 PMC6312258

